# Evaluation of a Single-Center Pilot Digital Inpatient Referral System: A Mixed-Methods Study

**DOI:** 10.7759/cureus.97728

**Published:** 2025-11-25

**Authors:** Saman Jeddi

**Affiliations:** 1 General Medicine, Basildon University Hospital, Mid and South Essex NHS Foundation Trust, Basildon, GBR; 2 Acute Medicine, The Queen Elizabeth Hospital King’s Lynn NHS Foundation Trust, King's Lynn, GBR

**Keywords:** clinical communication, clinician-led innovation, digital health, e-health, electronic referral systems, health informatics, hospital workflow, inpatient referral, machine learning in healthcare, quality improvement

## Abstract

Background: Effective and timely inpatient referrals are critical to maintaining patient safety, continuity of care, and efficient clinical decision-making within hospitals. At the Queen Elizabeth Hospital King’s Lynn, the long-standing “Greencard” paper system was widely used to manage inpatient referrals between specialties. However, such methods are vulnerable to illegibility, misplacement, delays, and lack of auditability, often leading to inefficiencies and fragmented communication. These limitations were further exposed during the coronavirus disease 2019 pandemic, when the need for remote, traceable, and secure communication increased across healthcare settings.

Methods: This study aimed to address these challenges: a bespoke electronic referral platform, termed the “e-Greencard” and formally known as E-Referral System for Hospital Admissions (ERSHA), was developed and piloted as a locally designed, in-house digital alternative. Created using open-source web technologies by a clinician, ERSHA aimed to provide a secure, auditable, and user-friendly referral workflow that could replace paper-based processes without reliance on external IT vendors. This pilot study evaluated the Phase 1 implementation of ERSHA across three inpatient specialties, cardiology, respiratory medicine, and neurology, using a mixed-methods approach combining referral analytics and user feedback.

Results: Over six months, 1,033 inpatient referrals were logged through ERSHA. Cardiology accounted for most submissions (625, 60.6%) and achieved the highest digital response rate (621/625, 99.4%). Respiratory medicine recorded 114 referrals with 99 replies (86.8%), while neurology recorded 35 replies from 294 referrals (11.9%), reflecting differing workflow integration. Overall, 755 of 1,033 referrals (73.1%) generated replies, rising to 97.4% when neurology was excluded. Survey feedback (n = 6) indicated good user satisfaction, with 83% rating the interface as easy to use and 100% supporting expansion to other specialties and outpatient referrals. However, the low formal response rate (5.9%) limits generalizability.

Conclusion: ERSHA demonstrated the feasibility and acceptability of a clinician-led, in-house digital referral platform developed without external IT support. The system was implemented in place of the traditional paper process, offering a secure and auditable digital workflow that clinicians perceived as more convenient, accessible, and transparent. Reported improvements were observational and based on user feedback rather than comparative metrics. Despite challenges such as single-server dependency and limited survey participation, the project illustrates the potential of locally driven innovation to support practical digitization within National Health Service hospitals. These findings should be viewed as exploratory and hypothesis-generating, with future iterations aiming to incorporate structured analytics, predictive features, and machine-learning-assisted triage to further enhance referral management and inpatient care.

## Introduction

Effective communication between clinical teams is a cornerstone of safe hospital care. Inpatient referrals in particular must be accurate, timely, and auditable. Historically, many National Health Service (NHS) hospitals have relied on paper-based methods, such as referral cards, which are prone to illegibility, delays, and loss. With increasing recognition of digital health's potential, especially after the coronavirus disease 2019 (COVID-19) pandemic, there has been a growing call for secure, locally adaptable tools that improve workflow efficiency [1]. However, implementing digital systems is often costly and reliant on third-party platforms or extensive IT infrastructure.

At The Queen Elizabeth Hospital King's Lynn NHS Foundation Trust, no dedicated digital solution existed to replace the paper-based Greencard referral system. In response, a grassroots digital referral tool called E-Referral System for Hospital Admissions (ERSHA) was developed using open-source web technologies.

Before the development of ERSHA, the inpatient referral process, similar to that in many NHS hospitals, relied almost entirely on manual, paper-based methods. At The Queen Elizabeth Hospital King’s Lynn, this took the form of a small A5-sized “Greencard” used to refer patients to specialists. While the Trust already used digital systems for other purposes, such as electronic discharge letters and radiology imaging, there was no unified or dedicated electronic platform for inpatient referrals between specialties. Paper methods, as widely documented, were fraught with inefficiencies, including illegibility, loss, misplacement, and miscommunication [2]. Common issues included incomplete or incorrect information, especially in outdated form sections, and delays, particularly when referrals were submitted late in the day and processed only the following morning. Once a card left the ward, tracking its status became difficult, and there was no consistent or reliable way to audit the process.

Digital systems for referrals were not widely used in the hospital where this trial took place. Although some specialties, such as vascular surgery and neurosurgery, used email systems or online databases based in other hospitals, this was, in general, rare. Phone calls were sometimes used for urgent surgical cases, and dermatology relied on emails, but the majority of medical specialties continued to use the paper-based method. The lack of a standardized digital system contributed to delays in patient care and complicated communication between generalists and specialists. The broader literature supports these observations; a systematic review highlighted how paper-based and poorly integrated digital referral systems contribute to inefficiencies and communication errors within hospitals, while electronic referral platforms can improve documentation quality, completeness, and overall referral appropriateness [3].

The need for a more streamlined and accountable referral system was clear, even though no formal research was conducted before the development of ERSHA. Based on anecdotal evidence and discussions with acute medicine consultants, it was evident that there was a gap in the current process. Furthermore, but not in the forefront, there was a desire to be able to clearly quantify this "unseen" workload, a need recorded to be of concern to exist in other specialties, too [4], and which electronic consultation systems have similarly documented by altering referral flows [5,6], aligning with established digital-maturity frameworks that assess the adoption and integration of health IT systems [7].

These needs drove the initial design and implementation of ERSHA. An international scoping review found that electronic referral systems are typically implemented at a single-hospital level and have demonstrated measurable improvements in efficiency, referral triage, and service accessibility, though their scalability and long-term sustainability remain limited [8]. To address these challenges, a bespoke electronic referral system, ERSHA, was designed and piloted as a digital alternative to the “Greencard” process. This study describes its development, implementation, and pilot evaluation within an acute hospital setting, using a mixed-methods approach to assess feasibility, uptake, and early outcomes.

## Materials and methods

The development of ERSHA

ERSHA began in a simple and experimental manner during the COVID-19 pandemic. Using fundamental web development skills, the initial prototype was built using standard HyperText Markup Language (HTML) and Cascading Style Sheets to replicate the fields from the original inpatient referral card. This form was the foundation of the software, but as the project developed, the design evolved. With time, PHP and MySQL were incorporated into the program, which enabled secure and encrypted referral data storage. Initially, templates for the PHP code were used, later being tailored to suit the needs of the system. Over time, what started as a single digital form expanded into a software with a structured MySQL database on an Apache Server.

Its development was not rigidly divided into clear-cut stages but rather followed a continuous evolution, with each new iteration building on the previous one. For the purposes of this article, the version trialed during the six-month pilot phase can be referred to as Phase 1. Programming software and frequent consultation with online resources for troubleshooting and learning were used to produce and develop ERSHA. A significant milestone in the development process was implementing Advanced Encryption Standard, 256-bit key, Cipher Block Chaining mode (AES-256-CBC) encryption to ensure data security, known to be used for patient data encryption [9], for which help from a senior experienced programmer was heavily dependent on. This encryption method was critical in safeguarding patient and referral data within the system.

Several key challenges arose during development, including the time required to learn coding, debugging, and refining both front-end and back-end components. A major functional breakthrough was the introduction of an email notification system that alerted referring clinicians when specialists responded, greatly improving communication and closing feedback loops. The notification system was designed to ensure timely communication between referring and receiving teams.

Each referral case ID was linked to the authorized user’s hospital email address. Whenever the case status changed, such as when a specialist reply was submitted, an automatic notification was sent via the hospital’s internal Microsoft Exchange server to the referring clinician and designated recipients (e.g., designated secretary). The accompanying “reply” feature, suggested by the supervising consultant, allowed specialists to respond directly within the system, further enhancing efficiency and auditability.

As ERSHA progressed toward its pilot phase, additional requirements were identified, such as a more user-friendly interface, secure data storage, and a print-friendly option for consultants preferring hard copies. The system was expanded to allow specialist secretaries to print referrals within specific date ranges and for resident and consultant doctors to access individual referrals securely using a hospital number combined with a randomly generated Personal Identification Number (PIN). These refinements ensured that the platform addressed the practical needs of both clinical and administrative users.

Trial setup

The trial of ERSHA was conducted over approximately six months, starting in April 2021 and concluding in October 2021. The goal of the trial was to evaluate how effectively the system could replace the existing paper-based referral card in three key specialties: cardiology, neurology, and respiratory medicine. One hundred two participants were involved in the trial, including resident and consultant doctors, as well as secretaries from the three participating specialties. ERSHA's Phase 1 version was principally developed between September 2019 and April 2021, with updates, maintenance, and bug fixes taking place throughout the trial period.

The platform was hosted on an internal Windows Server, following NHS guidance on local storage and key management recommendations. The system was piloted between April and October 2021 in acute medical units, with referrals made to cardiology, neurology, and respiratory teams (see Table 1 for timeline). The tool was web-accessible only within the hospital network. Although the hospital provided a server for hosting the software, there were concerns about its capacity to handle a large number of users. However, the internal Windows 10 Server with 8 GB of RAM and a fifth-generation Intel processor was able to manage the computing load without any issue when the server was running.

**Table 1:** Project timeline and key milestones

Stage	Date/period	Key milestones
Inception and conception	September-December 2019	Initial idea and design of secure hospital e-referral software; basic demo developed and shown to the supervising consultant
Early development	January-December 2020	Database schema created; referral form design; encryption and user login system implemented
Phase 1 pilot preparation	January-April 2021	Server hosted locally and tested; user access permissions defined; tutorial video created for clinicians; ethical approval obtained
Pilot goes live	April 27, 2021	First referrals entered (Cardiology, Neurology); respiratory referrals started April 30; regular maintenance, improvements, and monitoring initiated
Pilot ends	October 31, 2021	Total of 1,033 referrals processed by this date

A comprehensive technical and user manual was developed alongside ERSHA to ensure consistency, reproducibility, and operational continuity beyond the pilot team. Beyond outlining installation and configuration steps, it detailed user permissions, database administration guidance, and procedures for adding new specialties or updating contact emails. The manual also provided practical troubleshooting guidance, step-by-step instructions for data backup and recovery, and workflow guidance for administrative staff responsible for referral printing, auditing, and status updates. By documenting the entire system architecture and maintenance processes, the manual enabled independent replication of the platform and reduced reliance on the original developer. Completed in October 2020 following supervised verbal walkthroughs and an IT review, it served as both a training tool and a technical reference, facilitating smoother onboarding for new users and providing a framework for the long-term sustainability of the software.

Implementation

All participating resident doctors received structured training on using the system, supported by instructional videos and written guidance. Regular updates and troubleshooting notices were posted on the homepage to maintain user engagement. Access was controlled through a secure login system, and a physician associate in an administrative role oversaw account management and day-to-day maintenance. Consultants in the participating specialties were notified of the pilot and encouraged to provide replies directly within the platform, while secretarial staff printed referrals when preferred by individual consultants.

Software Architecture and Access Workflow

**Figure 1:**
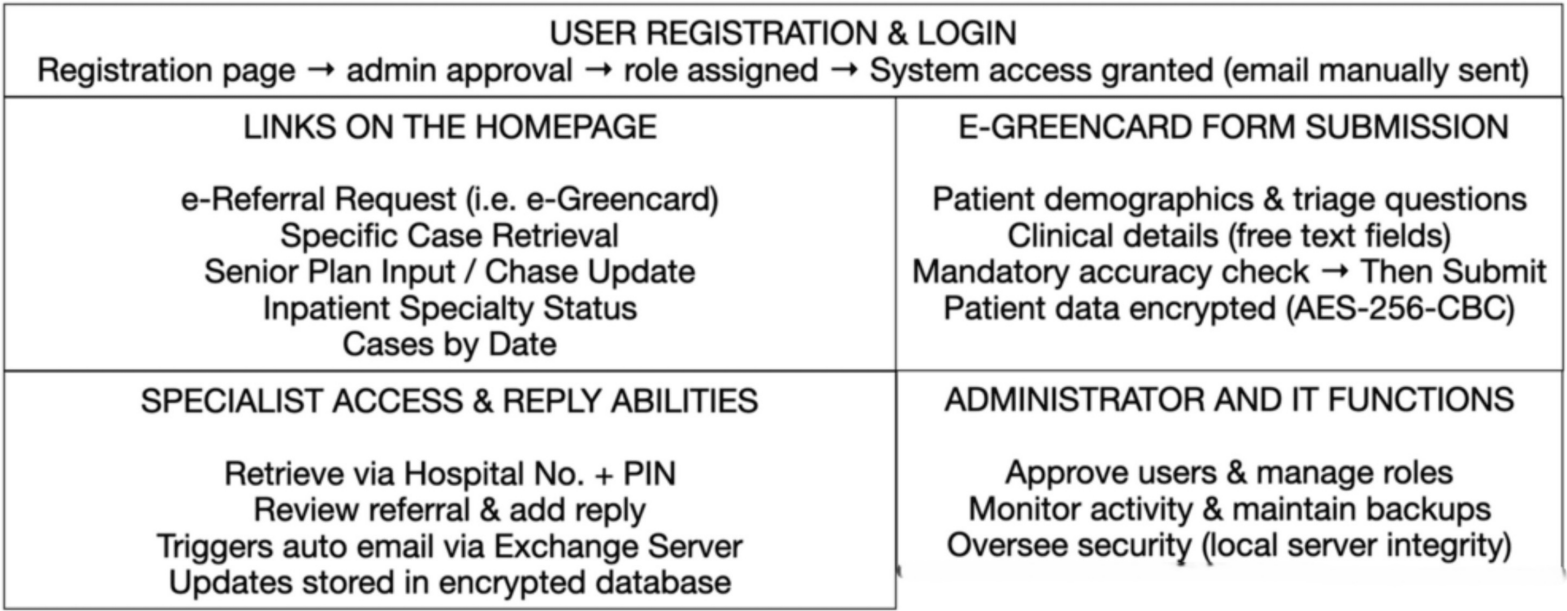
System architecture and functional workflow of the ERSHA platform The system operates through sequential modules: user registration and login, homepage navigation (with five functional links), referral form submission, specialist access and reply functions, and administrative oversight. Data security was maintained through AES-256-CBC encryption and local server hosting AES-256-CBC: Advanced Encryption Standard, 256-bit key, Cipher Block Chaining mode; exchange server: hospital internal mail system; ERSHA: E-Referral System for Hospital Admissions

The ERSHA platform functioned as a secure, web-based referral management system with three principal functional layers: user access, referral processing, and data retrieval (Figure 1). Users registered through the homepage by submitting their full name, hospital email, and a chosen password. Each registration request was verified by the system administrator against an authorized staff list, after which access was activated and a user role assigned. Access levels were stratified (resident/physician associate, senior, and administrator) to control data visibility and editing permissions. Once logged in, users were directed to a central dashboard with options to access the e-referral portal, view tutorial resources, or review recent updates. Automatic logout occurred after 15 minutes of inactivity to enhance security. Screenshots illustrating these stages of access and navigation are shown in Appendix 1.

The main homepage served as the functional hub of the system, with five primary modules: e-Referral Request (e-Greencard), Specific Case Retrieval, Senior Plan Input/Chase Update, Inpatient Specialty Status, and Cases by Date. Each module performed a distinct role in facilitating bidirectional communication between admitting and specialty teams. The e-Greencard module replicated the structure of the traditional paper referral form, capturing patient demographics, clinical details, and predefined triage questions (e.g., referral type, patient mobility, and diagnostic awareness). Free-text fields allowed users to enter the clinical question, history, relevant results, and investigation details. Before submission, a mandatory confirmation checkbox required verification of referral accuracy. Data were then securely transmitted to the internal database, encrypted using AES-256-CBC standards. Subsequent modules enabled retrieval of individual referrals using a hospital number and unique PIN, or bulk retrieval of cases by date and specialty (see Appendix 2).

Specialists could access assigned referrals, submit replies directly within the system, and trigger automated email notifications to referring clinicians via the hospital’s Microsoft Exchange server. Each referral was associated with a unique case ID linked to the referrer’s registered hospital email, allowing bidirectional communication while maintaining auditability. Administrators could monitor server activity and approve user accounts, while the IT team maintains regular encrypted backups.

During the pilot, an important enhancement was the introduction of the "availability status*"* feature, which displayed when certain specialties were temporarily unable to accept new referrals. Initially implemented as a manual process in Phase 1, this function was later refined in the planned Phase 2 development to allow automated adjustments based on consultant availability or staffing levels. Further details regarding this feature are discussed later in the Discussion section.

The final version used in the pilot incorporated multiple user-access levels, PIN-based referral tracking, email notifications, and role-specific functions such as printing, searching, and replying to referrals. System access was tiered to reflect clinical roles, allowing residents to submit and track referrals, senior clinicians to review and respond, and administrators to manage accounts. These are detailed in Table 2, along with the key features and user-level permissions incorporated into Phase 1 of the ERSHA platform.

**Table 2:** Summary of ERSHA Phase 1 functional features by user level Phase 1 was a web-based platform (HTML/PHP/MySQL) hosted on an internal Windows 10 server (8 GB RAM, Intel 5th Gen). Patient data security was ensured through AES-256-CBC encryption. The system operated on a three-tier access structure (Resident/Physician Associate → Senior/Secretary → Administrator) and generated automatic email notifications for both referral submission and specialist reply. Referral status could be viewed using a PIN combined with the hospital or NHS number. Audit functionality allowed date- and specialty-based searches for senior clinicians and administrative oversight ERSHA: E-Referral System for Hospital Admissions; HTML: HyperText Markup Language; SQL: Structured Query Language; AES-256-CBC: Advanced Encryption Standard, 256-bit key, Cipher Block Chaining mode

Feature/function	Resident doctors/physician associates (level 1)	Senior doctors/secretaries (level 2)	Administrator (level 3)
User access	Individual user accounts with secure login	Individual accounts for consultants, registrars, and designated secretaries	Master account with elevated privileges
Authentication	Login via username and password; system-generated PIN linked to patient hospital/NHS number for status tracking	Same access method as residents	Ability to activate, deactivate, or reset any user account
Referral creation	Create new inpatient referrals via a structured web form replicating the original “Greencard” fields	Can also create referrals if required	-
Referral form parameters	Patient demographics (name, date of birth, hospital/NHS number); referring consultant; consent confirmation; clinical summary (history, exam findings, investigations, past medical history, medications, management to date, working diagnosis); specific clinical question; vital parameters	Full access to view submitted referral data	Full access to database entries for maintenance/audit
Specialty selection	Select receiving specialty (cardiology, neurology, respiratory)	Full access	Full access
Referral tracking	Receive PIN and hospital number combination to check referral status (awaiting reply/replied)	Can check referral status by patient or date	Can monitor system-wide referral logs
Email notifications	Automatic email confirmation of referral submission and notification when a reply is received	Same email notifications upon reply submission	Receives system alerts and error logs
Reply function	View replies entered by specialists	Enter replies directly to referring clinician via integrated “Reply” feature (initially required manual patient entry; later automated button-based retrieval)	-
Search and audit tools	View personal referrals; limited search by own entries	Search referrals by date range, specialty, or status (awaiting/replied) for audit or review	Full database search and export functions
Printing functions	Print individual referrals for patient records if needed	Print referrals by date range for departmental use; secretaries are primarily responsible for printing if requested	Configure and maintain print templates
Specialty availability indicator	View specialty “availability status” on the submission page (whether the service is accepting new referrals)	Ability to toggle or update availability manually during pilot	Adjust or reset availability status settings globally
System access scope	Access within the secure hospital Intranet only	Same	Access to the MySQL database and the Apache server for system maintenance
Administrative controls	-	-	Activate accounts, change/reset passwords, update registered emails

Evaluation strategy

A mixed-methods approach was employed to evaluate the e-referral system. Quantitative data included the number and type of referrals, specialty response rates, and condition frequencies. Qualitative data were gathered through verbal feedback, posttrial surveys using best practice guidance [10], and issue reports. Survey participation was voluntary, and no patient-identifiable data were stored within the system. Governance approval was granted by the Trust’s Clinical Effectiveness and Audit Committee. This combination of activity data and user feedback mirrors established evaluation strategies for digital health adoption [7,11]. Users were able to provide feedback, ask questions, or offer suggestions through several online forms; however, the majority of input was received through direct contact with the project supervisor or the system administrator.

Evaluation criteria

As this was an exploratory pilot study, the primary focus was on assessing the feasibility, adoption, and usability of the ERSHA platform rather than predefined clinical performance thresholds. A priori success criteria were defined as follows: 1) Feasibility: successful deployment and continuous operation of a secure, locally hosted referral system throughout the six-month pilot without data loss; 2) Adoption and engagement: measured by referral volumes, specialty response rates, and diversity of referral types submitted; and 3) Perceived usability and satisfaction: assessed through posttrial survey responses evaluating ease of use, accessibility, and preference over the prior paper-based process.

Quantitative clinical outcomes (e.g., time-to-response) were not predefined at the outset, as the study’s primary intent was feasibility assessment. However, the system architecture included automatic timestamp logging for future analysis. During the pilot, a data capture error was identified that prevented consistent retrieval of these metrics. This functionality has since been corrected, and future iterations will formally evaluate process and clinical endpoints to enable comparative benchmarking.

Posttrial survey procedure

A self-designed posttrial survey (see Appendix 3) was distributed approximately one year after completion of the pilot to all 102 eligible users, including resident doctors, consultants, and physician associates. The questionnaire was developed specifically for this study to evaluate key aspects of the ERSHA platform, including usability, accessibility, workflow integration, and perceived impact on clinical communication. Responses were collected anonymously via an online form. Two reminder notifications were issued at approximately six-week intervals, resulting in three total invitations.

Qualitative Handling and Thematic Grouping

The posttrial survey contained a mix of Likert-scale and open-ended questions assessing usability, workflow integration, and perceived value of the ERSHA platform. Although a formal System Usability Scale was not used, a bespoke questionnaire was designed to capture similar constructs relevant to clinical workflow.

Responses were grouped conceptually into thematic domains derived from usability and implementation literature: 1) workflow fit and integration, 2) usability and cognitive load, 3) communication and responsiveness, and 4) perceived value and adoption. Free-text responses were reviewed descriptively, and representative comments were mapped to these domains to illustrate recurring perceptions. The thematic grouping of survey questions is summarized in Table 3.

**Table 3:** Thematic grouping of survey questions

Theme	Survey questions (summary phrasing)	Purpose/example insight
Workflow fit and integration	Duration of use; ease of layout; usefulness of the “specialty status” page; date-retrieval feature	Evaluates how well the system is integrated into daily clinical routines
Usability and cognitive load	Ease of registration; clarity of tutorials; system stability; ease of “senior reply” feature; implementation of feedback	Assesses ease-of-use, technical barriers, and user learning effort
Communication and responsiveness	Timeliness of senior replies; visibility of specialty availability	Measures communication flow and coordination between referrers and specialists
Perceived value and future adoption	Preference over paper/telephone; desire to expand to other or outpatient specialties	Gauges overall acceptance and intent for wider adoption
Open feedback (qualitative insight)	Free-text comments on improvements or suggestions	Captures unstructured opinions on workflow fit, usability, and engagement

## Results

Quantitative outcomes

Over the six-month trial, 1,033 referrals were submitted via ERSHA. Most were directed to cardiology (625, 60.6% of total referrals), followed by neurology (294, 28.4%) and respiratory medicine (114, 11.0%). Cardiology referrals were dominated by acute coronary syndromes (237, 37.9% of cardiology referrals), with additional demand for angina pectoris (90, 14.4%) and congestive cardiac failure (CCF; 66, 10.6%). Atrial fibrillation also accounted for 55 referrals (8.8%). Neurology referrals were primarily for seizures (91, 31.0% of neurology referrals), though migraine (24, 8.2%), epilepsy (24, 8.2%), stroke (13, 4.4%), and Parkinson’s disease (9, 3.1%) also featured. Respiratory referrals included chronic obstructive pulmonary disease (COPD) (22, 19.3% of respiratory referrals), pleural effusions (16, 14.0%), and suspected malignancy (12, 10.5%), as shown in Table 4.

**Table 4:** Summary of referral volumes, responses, and common conditions by specialty Percentages in parentheses refer to proportions within each specialty unless otherwise stated. The table summarizes referral volumes, replies, and response rates across three specialties during the six-month ERSHA pilot ACS: acute coronary syndrome; CCF: congestive cardiac failure; COPD: chronic obstructive pulmonary disease; NSTEMI: non-ST elevation myocardial infarction; ERSHA: E-Referral System for Hospital Admissions

Specialty	Total referrals, n (%)	Replies (n)	Response rate (%)	Top 3 conditions, n (%)
Cardiology	625 (60.6%)	621	99.4	NSTEMI/ACS 237 (37.9%); angina 90 (14.4%); CCF 66 (10.6%)
Neurology	294 (28.4%)	35	11.9	Seizures 91 (31.0%); migraine 24 (8.2%); epilepsy 24 (8.2%)
Respiratory medicine	114 (11.0%)	99	86.8	COPD 22 (19.3%); pleural effusion 16 (14.0%); suspected malignancy 12 (10.5%)
Total	1,033 (100%)	755	73.1%	-
Excluding neurology	739	720	97.4%	-

Response rates varied considerably between specialties. Cardiology demonstrated the highest engagement, with 621 of 625 referrals receiving a reply (99.4%). Respiratory medicine achieved 99 of 114 replies (86.8%). In contrast, neurology replied to only 35 of 294 referrals (11.9%), with feedback suggesting that teams continued to rely on phone communication or direct clinical review instead of the platform. Overall, 755 of 1,033 referrals (73.1%) generated replies via ERSHA, which rose to 720 of 739 referrals (97.4%) when neurology was excluded. Variation across specialties is common in early digital implementations and may relate to a combination of workflow practices and platform adaptation [7,12]. In most cases, responses directed users to updated clinical notes or via telephone rather than providing detailed plans within the system itself.

Across all specialties, the most frequently referred conditions were acute coronary syndromes (237, 22.9% of all referrals), seizures (91, 8.8%), angina (90, 8.7%), CCF (66, 6.4%), and atrial fibrillation (55, 5.3%). Less common but notable referrals included migraine (24, 2.3%), epilepsy (24, 2.3%), stroke (13, 1.3%), Parkinson’s disease (9, 0.9%), COPD (22, 2.1%), pleural effusion (16, 1.6%), and suspected malignancy (12, 1.2%).

Beyond the three most common diagnostic categories listed in Table 4, a broader range of conditions was recorded within each specialty, reflecting the diversity of inpatient referrals. In cardiology, while acute coronary syndromes and angina dominated, additional referrals included atrial fibrillation (55 cases), bradycardia, pericarditis, syncope, supraventricular tachycardia, acute and chronic kidney injury, and less frequent entries such as pericarditis, cellulitis, or COVID-19-related presentations. Myocardial infarction (possibly ST-elevation myocardial infarction) was occasionally recorded separately from non-ST-elevation myocardial infarction and may have contributed to minor under- or overrepresentation of acute coronary syndromes overall.

In neurology, referrals outside the major categories of seizures, migraine, and epilepsy included stroke (13 cases), Parkinson’s disease (nine cases), and smaller numbers of cases described as confusion/delirium, meningitis, collapse, or falls. Some entries used descriptive or uncertain terminology (e.g., likely seizure, background headache, progressive weakness), indicating variation in how referring clinicians documented working diagnoses. Rare cross-categorical terms such as sepsis or pneumonia were also encountered, perhaps reflecting multisystem presentations or the main organ being the cause of the sepsis (e.g., pulmonary sepsis).

In respiratory medicine, less frequent referral diagnoses comprised of obstructive sleep apnea, type 2 respiratory failure, asthma, pneumonia, pneumothorax, metastatic disease, fibrosis, and acute kidney injury. A few referrals also contained overlapping cardiac or systemic terms, such as CCF or hypertension. A summary of the different submissions overall is seen in Table 5.

**Table 5:** Summary of conditions recorded across specialties AF: atrial fibrillation; AKI: acute kidney injury; CKD: chronic kidney disease; COVID-19: coronavirus disease 2019; LRTI: lower respiratory tract infection; OSA: obstructive sleep apnea; STEMI: ST-elevation myocardial infarction; SVT: supraventricular tachycardia; TIA: transient ischemic attack; IECOPD: infective exacerbation of chronic obstructive pulmonary disease; COPD: chronic obstructive pulmonary disease; CCF: congestive cardiac failure

Cardiology	Neurology	Respiratory
AKI	Confusion/delirium	Asthma
Angina pectoris	Collapse (unspecified)	Hypertension (pulmonary)
AF	Epilepsy	IECOPD/COPD
Bradycardia (symptomatic)	Falls (unspecified cause)	LRTI
Cellulitis (with cardiac involvement/misclassified)	Headache (varied descriptors)	Malignancy
CKD	Meningitis	Metastasis
CCF	Migraine	OSA
COVID-19-related cardiac cases	Palsy (unspecified)	Pleural effusion
Myocardial infarction (STEMI classified)	Parkinson’s disease	Pneumonia
NSTEMI/ACS	Pneumonia (misclassified)	Pneumothorax
Pericarditis	Seizures	Pulmonary fibrosis
Sepsis	Sepsis	Respiratory failure (type II)
SVT	Stroke	Sepsis
Syncope/collapse	TIA	-

Seventeen referring consultants in the acute medical unit generated 82% (847/1,033) of all referrals, while the remaining 18% (186/1,033) were submitted under “Other,” likely representing senior resident doctors, registrars, or locum consultants not routinely based at the Trust. Nonparametric testing (Shapiro-Wilk, p = 0.002) indicated that referral numbers per consultant did not follow a Gaussian distribution. Therefore, data are summarized using the median and interquartile range (IQR). The median number of referrals per consultant was 29 (IQR: 17-83), as shown in Table 6.

**Table 6:** Distribution of referrals by consultant group Nonparametric distribution (Shapiro-Wilk, p = 0.002) justified the use of descriptive summaries only IQR: interquartile range; n: number of referrals

Doctor group	n	% of total referrals (n = 1,033)	Median (IQR)
Substantive consultants (n = 17)	847	82%	29 (18-74)
Other (e.g., registrars/locums)	186	18%	-

Qualitative data and feedback

Top Responses per Specialty

Across all specialties, the majority of senior replies directed clinicians to review patient notes for detailed management plans written rather than documenting these plans directly within the ERSHA database. Therefore, the reply was generally made after the patient was reviewed. Fewer than 10% of responses included explicit, structured guidance within the system itself, such as advising particular investigations, medication adjustments, or monitoring parameters. When specific replies were entered, they most commonly involved short, actionable recommendations, for example, “repeat ECG and troponin,” “arrange outpatient MRI,” or “request a CT Pulmonary Angiogram and inform me of the results.” These patterns appeared to vary by specialty, possibly influenced by differing workflow practices and how plans were customarily recorded in electronic records.

Survey Feedback

Respondents comprised physician associates (16.7%), resident doctors (50%), and senior doctors (33.3%), reflecting a representative cross-section of typical ERSHA users. Most respondents (66.7%) reported using ERSHA from the start of the trial. All respondents (100%) used the system for cardiology referrals, while half also used it for neurology and respiratory medicine. The majority (83.3%) agreed or strongly agreed that the video tutorials were helpful. Two-thirds (66.7%) strongly agreed that registration was easy, reflecting the simplicity of the account setup process. Most respondents (83.3%) strongly agreed that the layout was intuitive and user-friendly, confirming the platform’s design accessibility. Five of six respondents (83.3%) agreed or strongly agreed that specialist replies were timely, indicating satisfactory turnaround times within the system.

The majority (83.3%) found the specialty status page useful, validating its function as a workflow visibility tool. Five of six respondents (83.3%) reported experiencing minimal interruptions or software issues, supporting the system’s stability during the pilot. Only one respondent (16.7%) reported that their feedback had been implemented, while the majority (83.3%) did not submit feedback formally.

Among those eligible to comment on the "Senior reply" feature, responses were mixed; one rated it strongly disagree, one agree, and one strongly agree, likely reflecting both variation in familiarity and a small sample. All respondents (100%) supported extending ERSHA to additional specialties, reflecting strong enthusiasm for wider adoption. All respondents (100%) also supported expanding the system to include outpatient referrals, suggesting broad perceived applicability.

Most respondents (83.3%) preferred ERSHA to paper or telephone methods, reinforcing its usability advantage and perceived efficiency. No respondents provided additional written feedback. Two items on the questionnaire (“Senior reply feature was easy to use” and “Case retrieval by date was helpful”) were intended for senior users only. However, an extra response appears to have been entered for each, likely from a nonsenior participant. These were retained for transparency but interpreted cautiously in analysis.

A detailed item-level summary of all questionnaire responses is provided in Table 7. Furthermore, visual summaries of all survey responses, including Likert ratings and feature-specific feedback, are provided in** **Appendix 4.

**Table 7:** Detailed item-level responses from the posttrial survey Detailed summary of all responses to the posttrial feedback questionnaire distributed to 102 eligible users (six responses received; response rate 5.9%). Questions correspond to those shown in Appendix 3. Results are presented as counts and percentages of total respondents. Two items (“Senior reply feature was easy to use” and “Case retrieval by date was helpful”) were intended for senior users only; however, an extra response appears to have been entered for each, likely from a nonsenior participant. These minor discrepancies were retained for transparency but interpreted cautiously in analysis. Items rated on a 5-point Likert scale (from 1 = Strongly Disagree to 5 = Strongly Agree). Only responded options are shown ERSHA: E-Referral System for Hospital Admissions; SpR: Specialty Registrar; SHO: Senior House Officer

Survey question	Response options	n (%)
Role of respondent	Physician associate	1 (16.7%)
	Resident doctor (FY/SHO)	3 (50.0%)
	Senior doctor (SpR/consultant)	2 (33.3%)
Duration of use	From start of trial (April 2021)	4 (66.7%)
	2-3 months	1 (16.7%)
	3-4 months	1 (16.7%)
Specialties used for referrals	Cardiology	6 (100%)
	Respiratory medicine	3 (50%)
	Neurology	3 (50%)
Video tutorials were helpful	Disagree (2)	1 (16.7%)
	Agree (4)	1 (16.7%)
	Strongly Agree (5)	4 (66.7%)
Registration was easy and straightforward	Disagree (2)	1 (16.7%)
	Agree (4)	1 (16.7%)
	Strongly agree (5)	4 (66.7%)
Layout was easy to use	Neutral (3)	1 (16.7%)
	Strongly agree (5)	5 (83.3%)
Senior reply to my e-referrals was timely	Neutral (3)	1 (16.7%)
	Agree (4)	4 (66.7%)
	Strongly agree (5)	1 (16.7%)
Specialty status page was useful	Agree	5 (83.3%)
	Did not use	1 (16.7%)
Minimal interruptions or bugs	Neutral (3)	1 (16.7%)
	Agree (4)	1 (16.7%)
	Strongly agree (5)	4 (66.7%)
Feedback was implemented	Agree	1 (16.7%)
	Did not provide feedback	5 (83.3%)
Senior reply feature was easy to use (seniors only)	Strongly disagree (1)	1 (16.7%)
	Agree (4)	1 (16.7%)
	Strongly agree (5)	1 (16.7%)
Would like ERSHA for other specialties	Agree	6 (100%)
Would like ERSHA for outpatient referrals	Agree	6 (100%)
Case retrieval by date was helpful (for seniors/secretaries only)	Strongly disagree (1)	1 (16.7%)
	Neutral (3)	1 (16.7%)
	Strongly agree (5)	1 (16.7%)
Prefer ERSHA over paper/telephone referrals	Agree	5 (83.3%)
	Disagree	1 (16.7%)
Free-text comments provided	None	0 (0%)

Overall, the feedback suggested that ERSHA was perceived as user-friendly, stable, and functionally valuable. Respondents highlighted the clarity of the interface, ease of registration, and timeliness of senior replies as key strengths, aligning with the system’s design aims of accessibility and efficiency. The universal support for expansion to additional specialties and outpatient referrals indicates perceived scalability, although formal adoption will require broader evaluation and technical reinforcement.

System reliability

During the six-month pilot, the ERSHA platform maintained an overall estimated uptime exceeding 99%, with three recorded server-related incidents. These included one brief restart following a Windows security update (no downtime), one temporary outage of approximately one to two hours, and one major reinstall after a configuration corruption event. No referral or patient data were lost during any of these events. All data were protected through AES-256-CBC encryption and regular encrypted backups, verified after each restoration. The mean time to restore for the two outages requiring intervention was approximately 90 minutes, supported by the hospital IT department. Restore testing confirmed the integrity and completeness of all recovered records.

## Discussion

Principal findings and interpretation

The ERSHA pilot achieved its primary aim of demonstrating the feasibility of replacing a paper-based referral process with a functional, secure digital system. The high referral volume and strong response rate from cardiology suggest good engagement and acceptability among both senders and recipients. Similar findings were observed by Amer et al., who demonstrated that implementation of an e-referral system for inpatient surgical consults led to improved patient safety, better user experience, and reduced hospital stay durations, reinforcing the benefits of digitized intrahospital communication [13]. The system offered immediate improvements in accessibility, auditability, and standardization of referrals. Comparable design principles have been reported by Montellier et al., who described an electronic referral system for direct hospital admissions that improved communication pathways and coordination between referring physicians and hospital teams [14].

The quantitative survey findings, albeit limited by a low response rate, were broadly consistent with verbal feedback collected during the pilot. Most respondents rated the system positively for usability, reliability, and workflow integration, echoing the frequent informal reports of satisfaction received during the trial. The perceived timeliness of specialist replies also supported anecdotal impressions that digital referral tracking reduced delays compared with the previous paper-based system. Further feedback from the six-month trial of the ERSHA system was generally positive, with users noting that the digital format was more user-friendly and reliable than the former paper process. This perception was reinforced by posttrial survey responses, in which most participants agreed that the system layout was easy to use and that registration was straightforward. Respondents also reported that the specialty status feature was useful, reflecting overall satisfaction with the platform’s usability and workflow visibility.

The project is a clear example of clinician-led digital innovation, aligning with national NHS ambitions for localized health tech solutions [15]. Importantly, it was developed without external funding and used entirely freeware technologies. Challenges included the limited IT infrastructure (with no failover or remote access), low engagement in formal feedback mechanisms, and mixed uptake among different specialties. Server dependence remains a vulnerability, although it is mitigated by local IT support. Although the neurology team used the ERSHA platform to receive referrals, most responses were provided through traditional channels such as direct review or telephone discussion, rather than entered into the system itself. Future iterations will address this by incorporating registrar vetting and user prompts. Digital transformations are, nonetheless, very well documented to have implementation issues [12,16,17].

Pilot studies of evidence-based referral management platforms have also highlighted the importance of audit trails, structured referral content, and transparency between clinicians-features that were integral to the ERSHA design [18]. Certain practices proved essential for maintaining the system and supporting its users. Regular IT backups ensured that no patient data were lost during technical issues. IT support was invaluable in restarting and restoring the server when needed, minimizing disruptions. Finally, the use of an internal Exchange server enabled secure and reliable email notifications, which was a core function of ERSHA. However, because no baseline metrics were available for the prior paper-based system, any assumptions about improved efficiency should be regarded as observational and hypothesis-generating rather than confirmatory.

An exploration of the limitations and challenges

Analysis of Feedback and Response Rates

The posttrial feedback for ERSHA had a notably low response rate of 5.9%, despite three separate attempts to collect responses. This limited engagement may be attributed to the time lapse between the end of the trial and the request for feedback, which was sent roughly one year later. Many users may have since moved on to other systems or departments and no longer have active interaction with ERSHA, impacting their motivation to complete the survey. Additionally, general tendencies against survey participation in the healthcare environment may have contributed. Although a small sample size affects statistical representativeness, the qualitative insights from engaged users provide valuable direction for the future development of ERSHA.

Reality of Feedback Collection

Feedback during the trial was collected primarily through informal means, even though formal channels such as Google Forms (Google LLC, Mountain View, CA) were made available for bug reports and suggestions. In practice, most reports of bugs or improvement suggestions were delivered via secure internal email by the assigned administrator, who managed user accounts on the system. Approximately 10-15 issues were reported this way, and they were promptly addressed throughout the trial. Additionally, the supervising consultant provided regular feedback on how the system could be further improved.

Comparison With the Paper-Based System

Unfortunately, collecting baseline metrics to compare ERSHA to the previous paper-based referral process proved challenging. There were no formal data on response times or efficiency from the earlier system. However, anecdotal accounts suggested that the paper format sometimes led to delays, particularly when referrals were processed the following day. While the e-Greencard system recorded both referral submission and response times, a coding limitation during the pilot prevented consistent retrieval of these data. Nevertheless, feedback from the posttrial survey suggested that users generally perceived referral replies to be timely, indicating a positive experience with communication efficiency within the digital platform compared with traditional methods.

Technical and Operational Challenges

During the six-month pilot, several server-related incidents affected the continuity of the e-referral system. The first occurred in May 2021, when a routine Windows update prevented the server from restarting automatically. This was resolved with a manual reboot and caused no noticeable disruption. In September 2021, an unexpected server crash of unknown origin resulted in a temporary one-to-two-hour outage, which required IT assistance to restore functionality. Shortly after the trial concluded, a system failure occurred in October 2021, necessitating a complete reinstallation of the Apache/MySQL environment. This event coincided with the end of the pilot period. Table 8 summarizes the main server and technical events that occurred during the trial.

**Table 8:** Summary of server and technical events during the pilot phase

Date/period	Issue description	Cause/contributing factor	Resolution	Impact on the system
May 2021	The server failed to restart automatically after a scheduled Windows update	Windows update interrupted the Apache service startup sequence	Manual reboot performed by project lead; system restored without IT intervention	No data loss or disruption to users
September 2021	Unexpected temporary server outage lasting approximately 1-2 hours	Unknown system fault, possibly software conflict following Windows patch	IT department assistance; server restored after reconfiguration	Minimal user impact; system downtime ~2 hours
October 2021	System failure after another Windows Update; full reinstall required	Corruption of configuration files	Complete reinstallation of Apache/MySQL environment; data restored from backup	Temporary service loss; no patient data lost; trial subsequently also ended
Overall observation	Three server incidents were recorded during the six-month trial period	A combination of system updates and server configuration issues	Prompt IT support and regular backups ensured data integrity and continuity	Reinforced need for remote access and failover redundancy in future versions

Thanks to regular backups, no patient data was lost during any incident. These events highlighted the need for improved server reliability, particularly if ERSHA is to scale to additional departments. Troubleshooting sometimes required time-intensive reinstallation or reconfiguration of system components, emphasizing the importance of enhanced technical support and consideration of failover mechanisms in future iterations.

Participant Feedback and Insights

Some initial challenges did arise, such as occasional delays in account activation, which frustrated users attempting to access the system for the first time. Users also expressed a desire to expand the software to additional specialties beyond the initial trial areas. Feedback from the administrative side highlighted the need for more resources, such as video tutorials for different user roles, including specialized tutorials for administrators and secretaries. Although there was an introductory video, the need for more in-depth guides became apparent as users engaged with the system more extensively. Thus, further tailored videos were developed and shared to address these needs.

Administrator Feedback

The administrator shared general positive feedback, specifically from the respiratory team, and relayed specific feedback from consultants, who requested improvements for replying to individual referrals without needing to reenter patient information each time. This feedback led to the introduction of an interim solution during the trial, which allowed for updates and feature changes displayed on the website's homepage. Additionally, the administrator faced minor technical issues, such as some hospital PCs not displaying the system link properly and compatibility issues with the site URL on certain computers, which were swiftly resolved.

Diagnostic Phrasing Variability

The variability in diagnostic phrasing across referrals also revealed cross-specialty overlap, particularly in cases such as acute kidney injury and chronic kidney disease, which were often submitted under cardiology perhaps due to associated cardiorenal presentations. A few referrals also contained overlapping cardiac or systemic terms, such as CCF or hypertension. These observations suggest that while ERSHA captured a wide diagnostic spectrum, free-text variability and nonstandardized terminology limited precise condition bundling during postanalysis. Incorporating additional specialties, such as renal medicine or endocrinology, and introducing structured data entry fields or autoclassification tools could improve referral allocation accuracy and diagnostic granularity in future iterations.

Evaluation of the Manual

While a comprehensive technical and user manual was produced and reviewed by the IT team, its usability and uptake were not formally evaluated. Future iterations could assess how effectively such documentation supports user engagement and continuity beyond the development team.

Study Limitations

Several limitations identified throughout this discussion merit brief summarization. The pilot’s evaluation was constrained by a low posttrial survey response rate, which limited the representativeness of the feedback, even though verbal feedback during the trial was substantially richer. Baseline performance data from the prior paper-based referral system were unavailable, preventing direct efficiency comparisons. The platform was hosted on a single local server without redundancy, restricting scalability and technical resilience. Engagement varied across specialties, most notably in neurology, reflecting workflow differences rather than usability issues. Additionally, variability in diagnostic phrasing across referrals introduced minor ambiguity in categorization and analysis. Finally, although a comprehensive technical and user manual was produced and shared with IT staff, its usability was not formally evaluated. Collectively, these factors underscore that findings should be interpreted as descriptive and exploratory rather than comparative or inferential.

Future directions

Incorporation of Machine Learning

As healthcare datasets expand beyond what individual clinicians can manually interpret, machine learning has emerged as a fundamental technology for processing and drawing insight from large-scale clinical information [19,20]. While the present pilot’s dataset of 1,033 referrals across three specialties remains manageable by human analysis, scaling this system across departments or hospitals would rapidly increase its complexity. Future iterations of ERSHA could explore the use of machine learning to assist in identifying high-risk patients, predicting clinical deterioration, or prioritizing referrals for urgent review based on presenting features and trends in vital signs. Similar approaches have been discussed in recent literature as promising tools to support timely triage and improve decision-making [2,3].

Machine learning techniques have increasingly demonstrated potential to support clinical decision-making and workflow optimization across various domains of medicine [19]. In theory, future ERSHA development could integrate artificial intelligence (AI)-assisted vetting modules to review referral content before submission, helping to optimize triage and reduce consultant workload. These concepts remain prospective and untested within this project, but may enable faster response times and greater efficiency if validated in future evaluations. The unimplemented Phase 2, based on the existing patient data, predictive coding was inserted that gave probability management outcomes based on keywords in the patient history, combined with the working diagnosis. In the future, the efficacy of this in expediting patient management can be assessed and later developed into a more sophisticated model. Topol has argued that integrating human clinical reasoning with AI can create “high-performance medicine,” where AI assists clinicians in pattern recognition and complex triage while preserving human oversight and contextual judgment [20].

Future Parameters of Interest to Analyze

An area for future analysis involves quantifying referral and response time intervals. As briefly mentioned earlier, although both the referral submission time and the specialist response times were programmed to be recorded, a coding limitation meant that only the former could be reliably retrieved for analysis. As a result, the precise interval between referral submission and response could not be quantitatively calculated. Resolving this issue in future iterations would allow accurate measurement of response latency, enabling time-to-reply metrics to be correlated with specialty type, referral urgency, and clinical outcome.

That would then allow the evaluation of condition-specific response times to determine whether certain conditions receive faster replies, which could indicate priority areas or more efficient processes. Seasonal or time-based trends could be analyzed to identify peak periods for specific conditions, informing staffing or system updates. Additionally, a comparative analysis with paper-based referrals, where available, could assess improvements in handling high-demand conditions.

Maximizing Server Uptime

To enhance the robustness and reliability of the e-referral system, several recommendations were identified. Remote access capabilities, for example, providing a Trust-issued laptop with secure access, would allow for faster reconfiguration and troubleshooting in the event of server issues. Implementing a redundant failover server could further prevent disruptions, providing continuity of service and additional time for on-site reconfiguration if needed.

Common Communication Patterns

Analysis of referral content revealed that resident doctors primarily used general phrases such as “please review” or “kindly advise on management,” likely reflecting guidance from their seniors. This broad, nonspecific approach highlights an opportunity to improve how resident doctors are trained to ask targeted questions, which could enhance the efficiency of responses. Documentation practices were also noted: less than approximately 10% of management plans were documented directly in the system, suggesting that user habits or system design could be optimized to encourage more direct digital documentation. Informing clinicians on the efficient use of digital notes may improve data completeness and traceability. Finally, referral question quality could be enhanced through training resident doctors to frame specific, actionable queries. Clearer communication can reduce ambiguity and improve the accuracy and usefulness of responses.

Enrolling Advanced and Enhanced Features

Several advanced features were proposed and programmed for the planned Phase 2 to enhance system functionality. A later-developed audit feature would allow authorized senior clinicians to search and filter referrals by date, specialty, or referral status, improving transparency and traceability. In addition, a vetting module was designed to enable Specialty Registrars to review referrals before consultant review, providing an extra layer of triage and quality control. These tools were developed and tested in a non-live environment but were not deployed during the pilot. They remain ready for evaluation in a formal Phase 2 study, as outlined in Table 9.

**Table 9:** ERSHA phase 2: proposed and prepared new and enhanced functional features by user level Prepared, but not implemented, Phase 2 focused on workflow automation, predictive functionality, and usability enhancements, introducing a vetting stage for registrar review, a color-coded specialty-availability dashboard, a dynamic audit and search interface, a secure digital handover function, and an early “Likely Outcome” feature that generated probabilistic discharge or admission predictions from keyword patterns in referral data. Core security and hosting architecture from Phase 1 remained unchanged. Future Phase 3 development may extend these capabilities through structured diagnostic fields and machine-learning-based triage tools. The envisaged Phase 2 development also incorporates JavaScript and Python components to improve features, interface responsiveness, and database interaction CSS: Cascading Style Sheets; ERSHA: E-Referral System for Hospital Admissions

New/enhanced feature	Resident doctors/physician associates (level 1)	Senior doctors/registrars/secretaries (level 2)	Administrator (level 3)
Three-stage referral workflow	View expanded status (awaiting →vetting →replied); receive automated emails at each stage	Update status after vetting or consultant reply submission	Monitor overall status distribution for audit
Vetting module	See when referral under review; notified once vetted/rejected/updated with conditions	Registrar-level vetting of referrals for appropriateness with management feedback (e.g., “locally manage,” “request imaging”) or acceptance for senior review	Track numbers of vetted vs. pending referrals
Specialty availability dashboard	View color-coded traffic-light indicator on homepage (green = open; red = closed until specified time)	Toggle availability on/off; set date/time for reopening; add comments (e.g., “Contact via bleep/ward X”)	Override or reset availability settings system-wide
Enhanced audit and search tools	-	Interactive JavaScript-based dashboard to filter by date range, specialty, or status (identify outstanding actions)	Full database query and data export functions
Digital handover feature	Reassign responsibility for a referral to another clinician; successor receives subsequent email alerts	Approve or delegate handover within team for continuity of care	Record all handovers for audit and traceability
Improved interface	Enhanced front-end appearance with CSS and JavaScript, including optional display themes ranging from simple to colorful layouts for a more personalized user experience	Same enhanced interface	Same enhanced interface
Outcome-prediction tool	Lists likely outcomes in order of probability (e.g., admission, short-stay, discharge, or local management) based on keyword trends from the dataset	Could review and validate predicted outcomes for accuracy and clinical relevance; feedback used to refine the algorithm	Oversee database linkage and model updates for predictive analysis

Additional workflow improvements for a future Phase 2 include a color-coded specialty-availability dashboard and a secure digital handover function. The availability dashboard uses a traffic-light indicator to show when specific specialties are accepting new referrals, along with brief guidance on alternative contact routes when services are temporarily unavailable. The digital handover feature enables clinicians finishing their shift to transfer responsibility for an existing referral to another named colleague, ensuring that subsequent email notifications and updates are directed to the appropriate clinician. These enhancements further improve clarity, continuity, and communication between teams. Additionally, usability improvements also include the introduction of optional display themes, allowing users to personalize the interface and improve visual accessibility.

An additional prototype feature developed from the pilot dataset allows automatic generation of likely patient outcomes during referral entry. Using keyword matching mainly from the history and working diagnosis, the system presents estimated probabilities for outcomes such as admission, short-stay observation, or discharge. Although, like the other features above, this tool has not been deployed beyond internal testing, it demonstrates the potential for predictive analytics within the platform and informs the planned integration of machine-learning-based triage in a further-in-the-future Phase 3.

## Conclusions

The ERSHA pilot demonstrated the feasibility and potential value of a clinician-led, in-house electronic referral platform within a busy acute hospital. Developed without external IT or commercial support, it temporarily replaced a traditional paper-based process with a secure, auditable, and user-friendly digital workflow. High utilization and strong engagement from clinicians suggest good acceptability and alignment with NHS goals for cost-effective digitization. Feedback from users, though limited in scope, indicated that the digital system was perceived as accessible, reliable, and adaptable, supporting its feasibility for wider clinical application. A subsequent Phase 2 development aims to expand system capability through registrar-level vetting, a color-coded specialty-availability dashboard, an interactive audit tool, a secure digital handover feature, visual interface enhancements, and a prototype outcome-prediction tool.

Multiple challenges, including server reliability, variable specialty engagement, and limited formal feedback, emphasize the need for greater technical resilience, timely data collection, and tailored user training to support scalability. Further development (i.e., a further-in-the-future Phase 3) could explore structured diagnostic fields, cross-specialty expansion, and integration of machine-learning tools for predictive triage and risk stratification. Given the absence of comparator data and formal efficiency metrics, these results should be viewed as descriptive and hypothesis-generating rather than confirmatory. Even so, the ERSHA project highlights how local, clinician-led innovation can drive practical digitization within NHS hospitals and may provide a replicable framework for future e-referral solutions. The author welcomes discussion and collaboration regarding potential adaptation or implementation of ERSHA in other hospital or clinical settings.

## Figures and Tables

**Figure 2:**
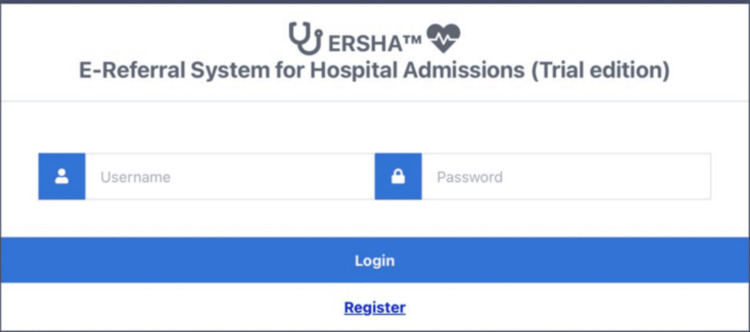
Login page screenshot The initial login interface, where users enter their hospital email and password to access the ERSHA platform. Registration also begins from this page ERSHA: E-Referral System for Hospital Admissions

**Figure 3:**
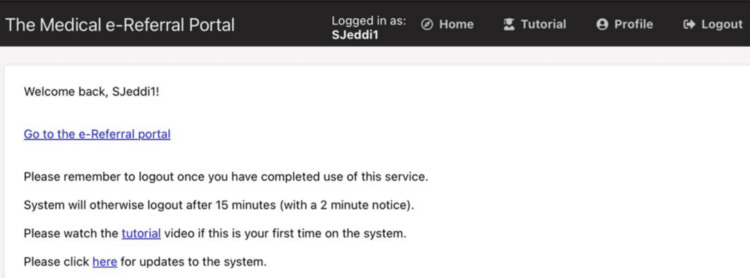
Welcome page after login screenshot User landing page displaying a personalized welcome message and navigation buttons linking to the e-Referral Portal, tutorial video, and system update information includes a security notice and an automatic logout feature, after 15 minutes of inactivity

**Figure 4:**
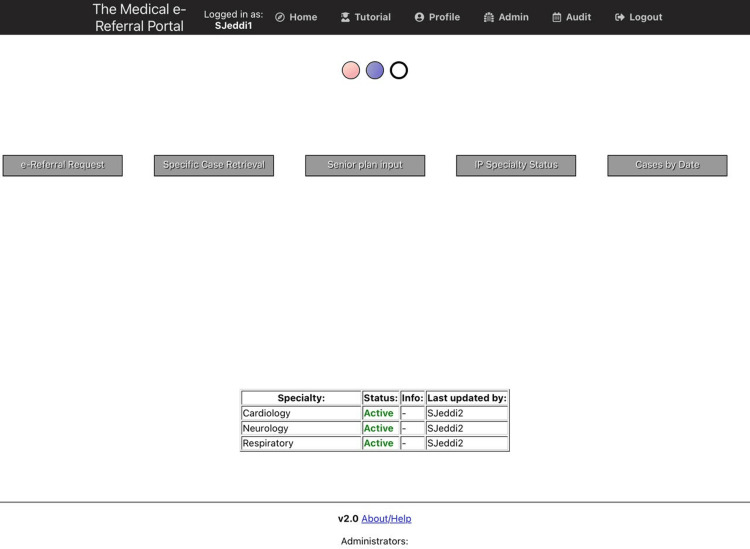
Homepage dashboard screenshot The central navigation hub of ERSHA features key modules: e-Referral Request (e-Greencard), Specific Case Retrieval, Senior Plan Input/Chase Update, Inpatient Specialty Status, and Cases by Date. Includes a top menu bar with Home, Tutorial, Profile, and Logout options. Note that the screenshot is from the updated "Version 2.0" that was aimed to be used in a future "Phase 2" of the software run. Therefore, it includes a three-circle feature that changes the theme for the user. Furthermore, the specialty status is updated, as explained in the Discussion, as well as a horizontal navigation button addition called "Audit" ERSHA: E-Referral System for Hospital Admissions

**Figure 5:**
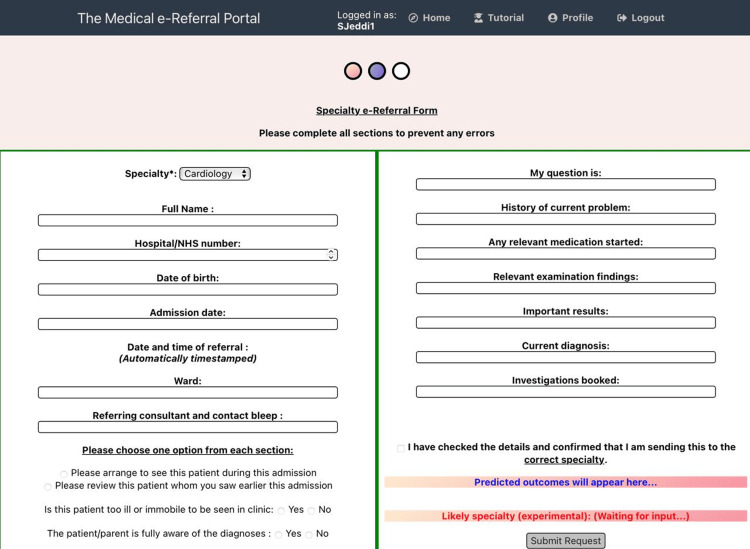
Screenshot of e-Greencard referral form Digital referral form replicating the structure of the traditional paper “Greencard.” Collects patient identifiers, referral type, clinical details, and has a verification checkbox before submission to the secure, encrypted database. Note here also that there is a three-button theme selection button, whereby, in this case, the first design is selected; hence, the background is of a pale red/pink color. Furthermore, the mentioned feature of an "Outcome Prediction Tool" is seen in the bottom right-hand corner of the form before submission. Finally, a very experimental feature of "Likely Specialty" prediction based on the free text input from the data gained in the Pilot-Study is also seen

**Figure 6:**
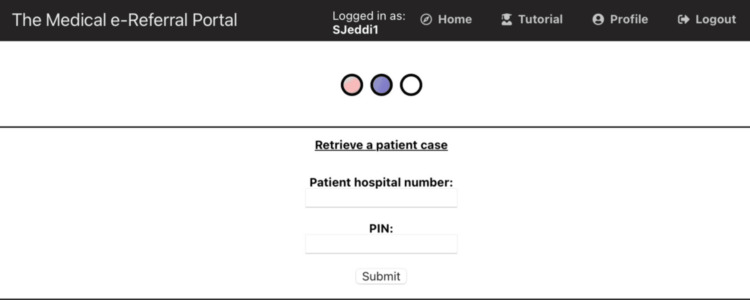
Retrieve inpatient case page screenshot Form allowing users to retrieve a specific referral by entering the hospital number and unique PIN, displaying the full referral record in a formatted “letter-style” layout on the subsequent page

**Figure 7:**
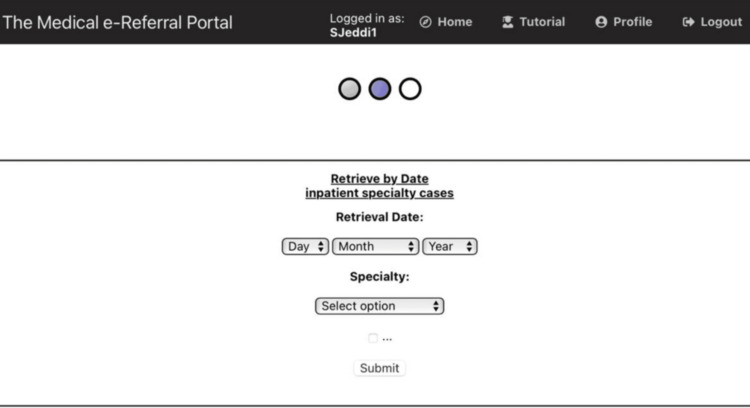
Retrieve by date page screenshot Administrative tool accessible to senior users enabling bulk retrieval of all referrals for a selected specialty and date range, supporting audit and reporting functions

**Figure 8:**
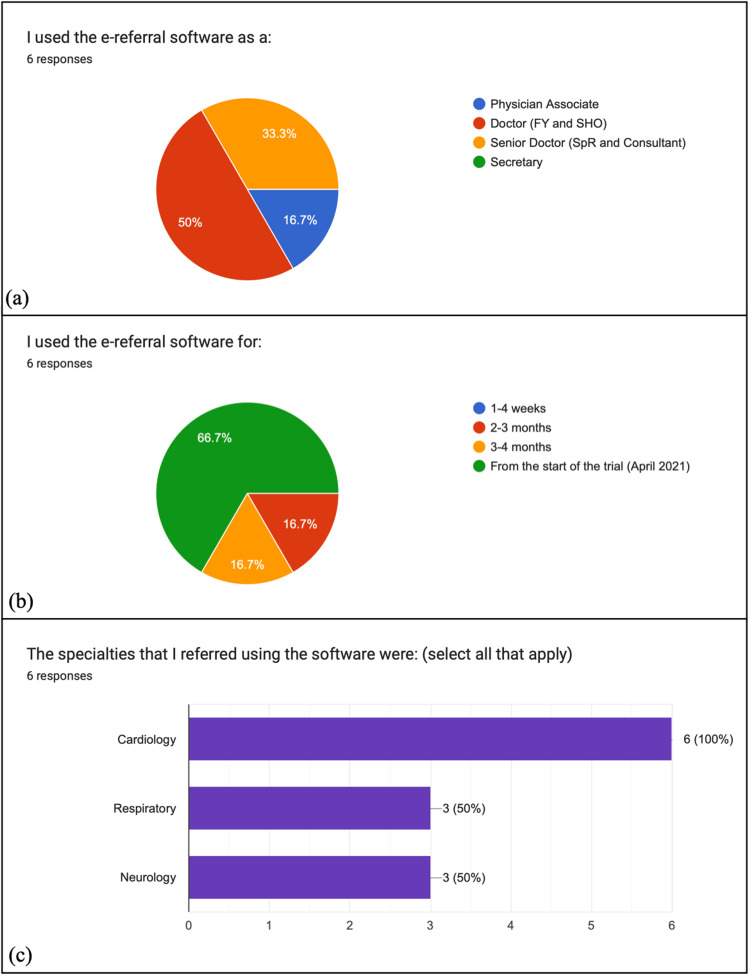
Survey response summary I: user roles, duration of use, and specialty coverage (a) Pie chart showing the distribution of respondent roles in the posttrial feedback survey. Colors correspond to user categories: blue = Physician Associate, red = Doctor (FY/SHO), yellow = Senior Doctor (SpR/Consultant). No responses were received from secretaries (green). (b) Pie chart showing the self-reported length of exposure to the ERSHA software during the pilot period. Colors correspond to usage time: blue = 1-4 weeks, red = 2-3 months, yellow = 3-4 months, green = from the start of the trial (April 2021). (c) Bar chart displaying the number of respondents selecting each specialty used for referrals (multiple selections allowed). The X-axis represents the number of respondents (0-6); the Y-axis lists the three pilot specialties (Cardiology, Neurology, Respiratory Medicine)

**Figure 9:**
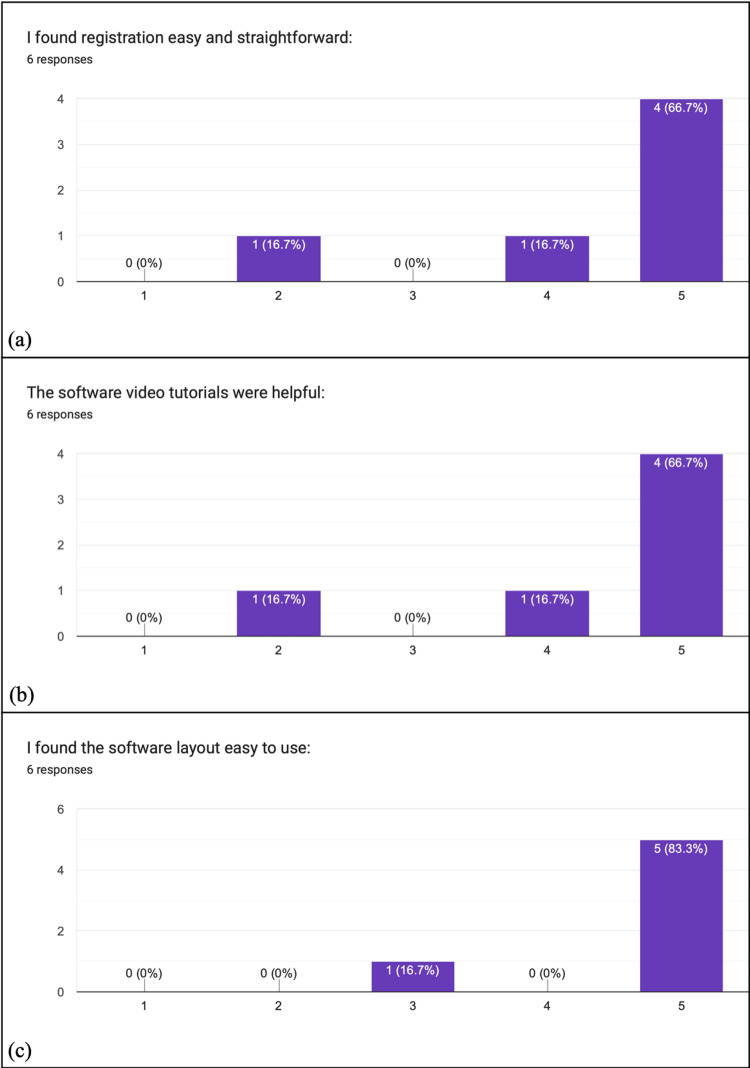
Survey response summary II: ease of registration, tutorial helpfulness, and layout usability (a) Bar chart showing responses to the statement “I found registration easy and straightforward.” The X-axis represents Likert-scale ratings from 1 (Strongly Disagree) to 5 (Strongly Agree); the Y-axis shows the number of respondents selecting each rating. (b) Bar chart showing responses to “The software video tutorials were helpful.” Axes are as above. (c) Bar chart showing responses to “I found the software layout easy to use.” Axes are as above

**Figure 10:**
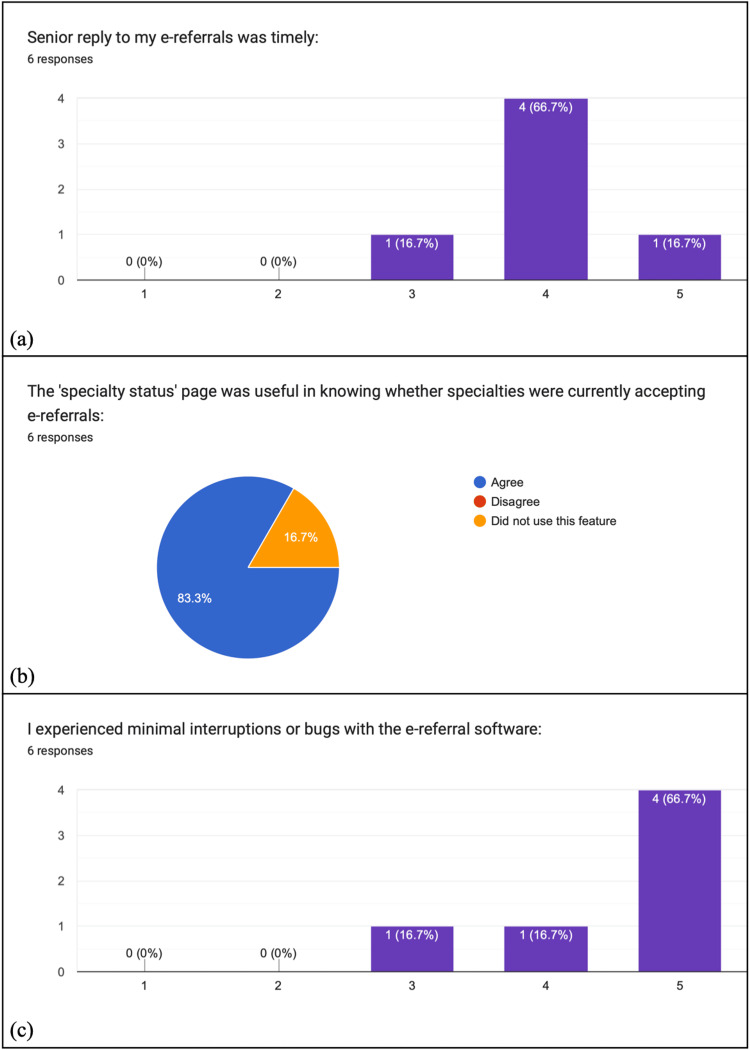
Survey response summary III: timeliness, feature utility, and system reliability (a) Bar chart showing responses to the statement “Senior reply to my e-referrals was timely.” The X-axis represents Likert-scale ratings from 1 (Strongly Disagree) to 5 (Strongly Agree); the Y-axis shows the number of respondents selecting each rating. (b) Pie chart showing responses to “The specialty status page was useful in knowing whether specialties were currently accepting e-referrals.” Blue = Agree, red = Disagree, and yellow = Did not use this feature. Percentages are shown within each segment. (c) Bar chart showing responses to “I experienced minimal interruptions or bugs with the e-referral software.” The X-axis represents Likert-scale ratings from 1 (Strongly Disagree) to 5 (Strongly Agree); the Y-axis shows the number of respondents selecting each rating

**Figure 11:**
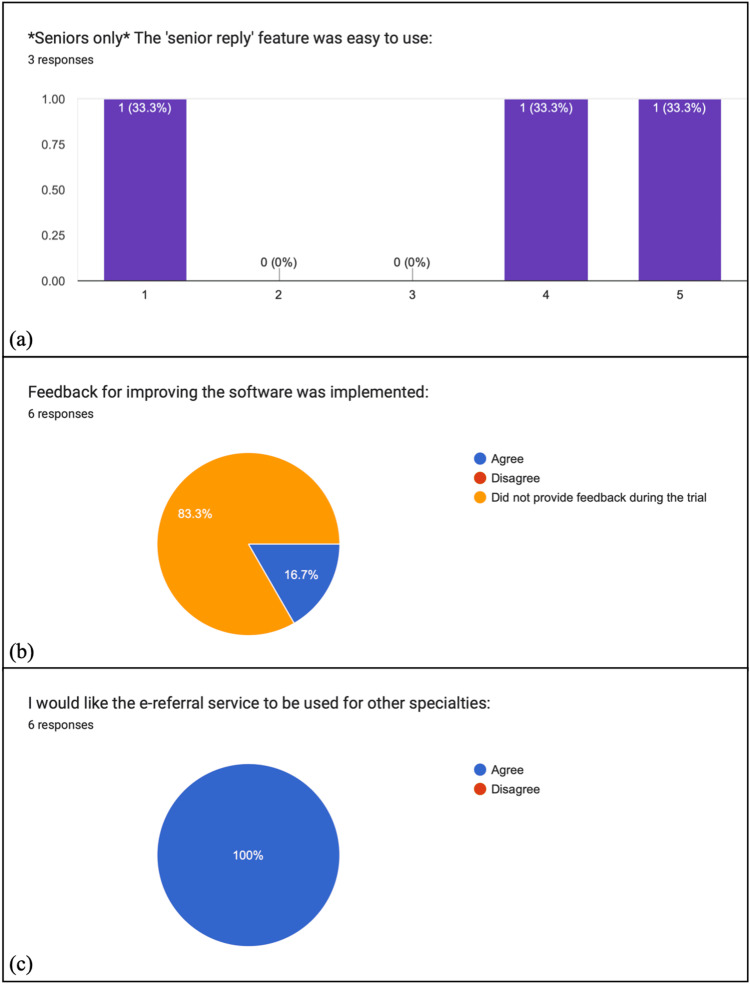
Survey response summary IV: senior feature usability, feedback implementation, and interest in expansion (a) Bar chart showing responses to “The senior reply feature was easy to use.” The X-axis represents Likert-scale ratings from 1 (Strongly Disagree) to 5 (Strongly Agree); the Y-axis shows the number of respondents selecting each rating. This item was intended for senior users only (n = 3). One additional response may have been entered in error, as only two senior respondents were confirmed in the overall survey sample. (b) Pie chart showing responses to “Feedback for improving the software was implemented.” Blue = Agree, red = Disagree, and orange = Did not provide feedback during the trial. (c) Pie chart showing responses to “I would like the e-referral service to be used for other specialties.” Blue = Agree; red = Disagree

**Figure 12:**
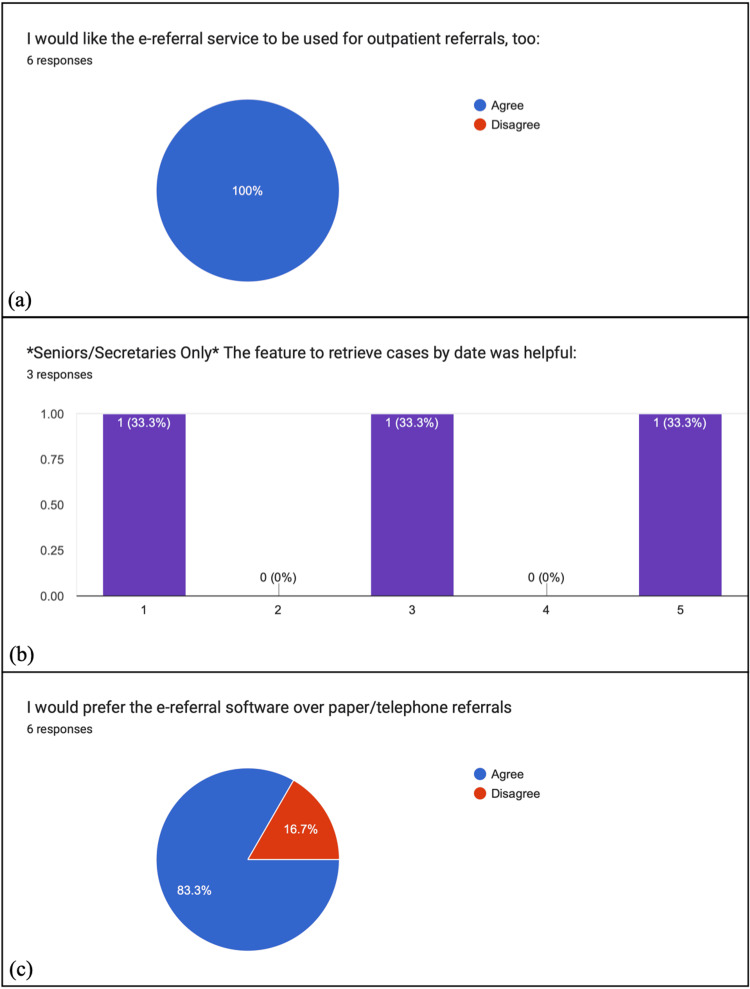
Survey response summary V: future use, advanced feature utility, and overall preference (a) Pie chart showing responses to “I would like the e-referral service to be used for outpatient referrals too.” Blue = Agree; red = Disagree. (b) Bar chart showing responses to “The feature to retrieve cases by date was helpful.” The X-axis represents Likert-scale ratings from 1 (Strongly Disagree) to 5 (Strongly Agree); the Y-axis shows the number of respondents selecting each rating. This question applied only to senior and secretary users. As only two senior respondents participated, one additional response may have been entered in error and should be interpreted with caution. (c) Pie chart showing responses to “I would prefer the e-referral software over paper/telephone referrals.” Blue = Agree; red = Disagree

## Appendices

Appendix 1

Appendix 2

Appendix 3

**Table 10:** Posttrial ERSHA feedback questionnaire Complete list of items included in the posttrial survey distributed to 102 eligible users following the six-month pilot phase of the ERSHA platform. The questionnaire assessed usability, functionality, workflow integration, and user satisfaction through Likert-scale, categorical, and open-response questions. Note that none of the feedback submissions gave free-text input FY: Foundation Year; SHO: Senior House Officer; SpR: Specialty Registrar; ERSHA: E-Referral System for Hospital Admissions

Question no.	Question/item	Response options
1	Email	Open text (required)
2	I used the e-referral software as a:	Physician Associate/Doctor (FY or SHO)/Senior Doctor (SpR or Consultant)/Secretary
3	I used the e-referral software for:	1-4 weeks/2-3 months/3-4 months/From the start of the trial (April 2021)
4	The specialties that I referred using the software were:	Cardiology/Respiratory/Neurology (Select all that apply)
5	The software video tutorials were helpful:	5-point Likert scale (1 = Strongly Disagree → 5 = Strongly Agree)
6	I found registration easy and straightforward:	5-point Likert scale (1 = Strongly Disagree → 5 = Strongly Agree)
7	I found the software layout easy to use:	5-point Likert scale (1 = Strongly Disagree → 5 = Strongly Agree)
8	Senior reply to my e-referrals was timely:	5-point Likert scale (1 = Strongly Disagree → 5 = Strongly Agree)
9	The “specialty status” page was useful in knowing whether specialties were currently accepting e-referrals:	Agree/Disagree/Did not use this feature
10	I experienced minimal interruptions or bugs with the e-referral software:	5-point Likert scale (1 = Strongly Disagree → 5 = Strongly Agree)
11	Feedback for improving the software was implemented:	Agree/Disagree/Did not provide feedback during the trial
12	Seniors only: The “senior reply” feature was easy to use:	5-point Likert scale (1 = Strongly Disagree → 5 = Strongly Agree)
13	I would like the e-referral service to be used for other specialties:	Agree/Disagree
14	I would like the e-referral service to be used for outpatient referrals, too:	Agree/Disagree
15	Seniors/Secretaries only: The feature to retrieve cases by date was helpful:	5-point Likert scale (1 = Strongly Disagree → 5 = Strongly Agree)
16	I would prefer the e-referral software over paper/telephone referrals:	Agree/Disagree
17	Any other feedback, improvements, suggestions, or comments:	Open text (optional)

Appendix 4
